# Ecological vaccination: A strategy to prevent zoonotic spillover from bats

**DOI:** 10.1126/sciadv.aec0269

**Published:** 2026-03-11

**Authors:** Hongyue Li, Fei Yuan, Yanfeng Yao, Jinglin Wang, Chunzheng Li, Ye Liu, Jinmei Yang, Zhongyang Zhang, Jiandong Liu, Jiankai Liu, Zhenxing Yang, Wanzhu Jin, Dan Wen, Chao Shan, Aihua Zheng

**Affiliations:** ^1^ State Key Laboratory of Animal Biodiversity Conservation and Integrated Pest Management, Institute of Zoology, Chinese Academy of Sciences, Beijing 100101, China.; ^2^ State Key Laboratory of Virology and Biosafety, Wuhan Institute of Virology, Chinese Academy of Sciences, Wuhan 430071, China.; ^3^ Center for Biosafety Mega-Science, Wuhan Institute of Virology, Chinese Academy of Sciences, Wuhan 430071, China.; ^4^ Yunnan Key Laboratory of Cross-Border Infectious Disease Prevention and New Drug Development, Public Health, Kunming Medical University, Kunming 650500, China.; ^5^ College of Life science, Henan Normal University, Xinxiang 453003, China.; ^6^ Jilin Heyuan Bioengineering Co. Ltd., Jilin 138000, China.; ^7^ Beijing Minhai Biotechnology Co. Ltd., Beijing 102629, China.; ^8^ Yunnan Tropical and Subtropical Animal Viral Disease Laboratory, Yunnan Animal Science and Veterinary Institute, Kunming 650500, China.; ^9^ Key Laboratory of Animal Ecology and Conservation Biology, Institute of Zoology, Chinese Academy of Sciences, Beijing 100101, China.; ^10^ University of the Chinese Academy of Sciences, Beijing 100039, China.; ^11^ CAS Center for Excellence in Biotic Interactions, University of Chinese Academy of Sciences, Beijing 100049, China.

## Abstract

Bats serve as critical reservoirs of zoonotic pathogens but also play essential ecological roles. To mitigate spillover risks without harming bat populations, we developed a multiroute vaccination strategy using recombinant vesicular stomatitis virus (rVSV)–based vaccines. Vaccine-carrying mosquitoes delivered rVSV-based rabies and Nipah vaccines, conferring protection in rodent and bat models. Under simulated natural conditions, cohabitation with vaccine-carrying mosquitoes elicited strong immune responses in bats, supporting feasibility beyond laboratory settings. As a complementary approach, saline traps exploiting bats’ mineral-seeking behavior achieved comparable immune protection through oral vaccination. Together, these results demonstrate a flexible, ecology-informed vaccination platform for immunizing wild bats, offering a scalable strategy to reduce zoonotic spillover risk while supporting bat conservation.

## Teaser

Harnessing natural behaviors to vaccinate wild bats reduces zoonotic spillover risk while supporting conservation.

## INTRODUCTION

Bats (Chiroptera), representing ~22% of all mammalian species, are natural reservoirs for a wide range of zoonotic viruses, including coronaviruses, rhabdoviruses, and paramyxoviruses (*1*–*3*). Their unique physiological and immunological traits enable them to harbor pathogens without showing clinical symptoms, making them critical players in the emergence of infectious diseases (*4*–*6*). Over the past decades, bat-borne viruses have caused major outbreaks, including Ebola, severe acute respiratory syndrome (SARS), Middle East respiratory syndrome, and COVID-19, highlighting the pressing need for strategies to mitigate spillover risks (*7*–*10*). Immunizing bats, particularly those in the wild, could serve as a proactive measure to decrease zoonotic transmission at its source. However, achieving this goal presents substantial challenges due to the wide geographic distribution, diverse diets, and large colony sizes of bat populations.

Conventional vaccination methods, such as intramuscular injections, topical applications, or oral baits, are impractical for large-scale implementation in wild bats. Previous attempts using inactivated rabies vaccines or recombinant viral vectors have yielded limited success and scalability (*11*–*14*). While experimental vaccines have been tested on captive bats, none have been effectively applied in wild populations. Additionally, bat culling, a strategy used in Latin America to control rabies, has proven counterproductive, potentially exacerbating disease transmission (*15*). These challenges call for innovative vaccination strategies that align with bats’ natural behaviors and ecological dynamics.

The rabies virus (RABV), a member of the family Rhabdoviridae, is primarily hosted by carnivorous mammals such as foxes and wolves. It has also been isolated from various bat species, including *Lasionycteris noctivagans*, *Perimyotis subflavus*, and *Murina leucogaster*. Domestic dogs frequently acquire rabies through contact with infected wildlife, posing a serious public health threat (*16*, *17*). While vaccination campaigns in the 20th century successfully controlled rabies in mesocarnivores, such as foxes (*Urocyon cinereoargenteus* and *Vulpes vulpes*) and raccoons (*Procyon lotor*), the virus continues to persist in bat reservoirs, leaving a critical gap in rabies control (*18*, *19*). Similarly, the Nipah virus (NiV), a paramyxovirus with a high mortality rate in humans, is primarily hosted by frugivorous bats: *Pteropus* bats. NiV spillover occurs through two main routes: intermediate hosts, such as pigs and horses, or direct human consumption of date palm sap contaminated by bat saliva or urine (*20*, *21*). Current control efforts focus on interrupting these interspecies transmission chains by preventing contact between bats and livestock or limiting bat contamination of date palm sap (*22*). For both rabies and NiV, directly targeting bats, the natural reservoirs of these viruses, is crucial to preventing spillover events and mitigating public health risks.

To overcome the challenges of vaccinating wild bats, we developed an innovative immunization strategy using a live vaccine based on recombinant vesicular stomatitis virus (VSV), a versatile arbovirus capable of infecting both insects and mammals (*23*, *24*). By leveraging the natural interactions between bats and mosquitoes, we designed two delivery approaches: using mosquitoes as vaccine carriers and using saline traps to attract bats for oral vaccination. Mosquitoes, which feed on bats and are also prey for them, serve as natural vectors for vaccine delivery (*25*–*28*). Simultaneously, the saline trap exploits the bats’ mineral-seeking behavior (*29*–*31*), providing a practical and scalable method for oral vaccine administration.

In this study, we evaluated the efficacy of rVSV-based vaccines against RABV and NiV in rodent and bat models. Both mosquito-mediated and oral delivery methods successfully induced protective immunity, demonstrating the feasibility of these strategies. This study represents a significant step forward in both zoonotic disease control and bat conservation, offering an innovative, ecologically informed solution to a pressing global health challenge.

## RESULTS

### Generation of mosquito-delivered RABV vaccine

**Fig. 1.**
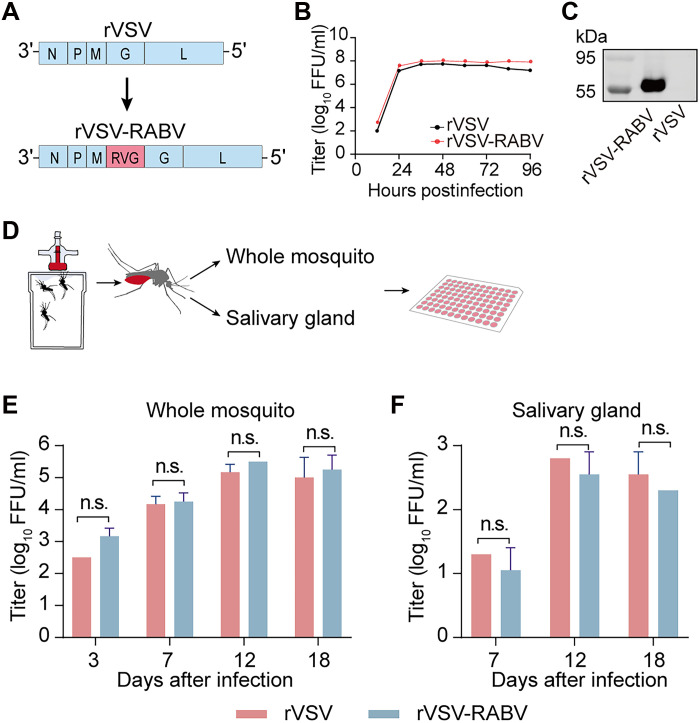
Characterization of rVSV-RABV and its propagation in *Ae. aegypti*. (**A**) Schematic diagram of rVSV-RABV, which was engineered by inserting the open reading frame of the RABV *G* gene (RVG) between the VSV matrix (*M*) and glycoprotein (*G*) genes in the vector. (**B**) Growth kinetics of rVSV-RABV and rVSV in Vero cells. (**C**) Western blot analysis of purified rVSV-RABV and rVSV from supernatants, probed with an antibody against RABV G protein. (**D**) Schematic illustration of mosquito infection via blood feeding with either rVSV-RABV or rVSV. Viral titers in whole mosquitoes (**E**) and salivary glands (**F**) were assessed at indicated days post–blood feeding by focus-forming assay. Error bars indicate SD of the mean. Statistical significance was determined using the unpaired two-tailed Student’s *t* test. Above data are representatives of three independent experiments. n.s., not significant.

To develop a rVSV-vectored vaccine delivered by mosquitoes, we inserted the RABV *G* gene between the *M* and *G* genes of VSV carrying M51 deletion in M protein (*32*) (rVSV-RABV) (Fig. 1A). The rVSV-RABV was amplified in Vero cells with a titer of 10^8^ focus-forming units (FFU)/ml, similar to the parent virus rVSV (Fig. 1B). The incorporation of RABV glycoprotein G in recombinant rVSV-RABV was confirmed by Western blot (Fig. 1C).

We next evaluated the infection of lab-adapted *Aedes aegypti* mosquitoes with rVSV-RABV via blood feeding (Fig. 1D). Both rVSV-RABV and control rVSV were detected in whole mosquitoes at day 3 postfeeding, which persisted for at least 18 days, reaching a peak titer of ~3.1 × 10^5^ FFU/ml. The rVSVs were also detected in the salivary glands by day 7, which persisted for at least 18 days, with a peak titer of 4.2 × 10^2^ FFU/ml (Fig. 1, E and F). The levels of rVSV-RABV in both whole mosquitoes and salivary glands were comparable to those of rVSV. These findings demonstrate that rVSV-RABV effectively proliferates in *Ae. aegypti* and is disseminated to their saliva.

To minimize the potential ecological safety risks associated with releasing lab-adapted mosquitoes into the environment, we treated rVSV-infected *Ae. aegypti* with x-ray irradiation, as previously described (*33*). A dose of 40 gray (Gy) completely sterilized mosquitoes without obvious effects on their survival, engorgement, or salivary vaccine titer (fig. S1). Furthermore, rVSV-infected female mosquitoes neither transmitted the virus to their mating partners nor exhibited vertical transmission to their offspring, effectively eliminating the risk of environmental virus leakage (fig. S2).

We further evaluated the safety of rVSV in its natural host pigs. Following high doses of 10^8^ FFU intradermal injections, none of the Bama pigs showed signs of weight loss (fig. S3A). Viruses were not detected in various tissues or secretions, and all animals seroconverted, except for one pig that showed detectable viral RNA in tongue tissue (fig. S3, B to E). These results indicate that transient replication of rVSV in its livestock host does not cause significant clinical illness and viral shedding.

### Mosquito-mediated rVSV-RABV vaccination elicits protective immunity in mice

**Fig. 2.**
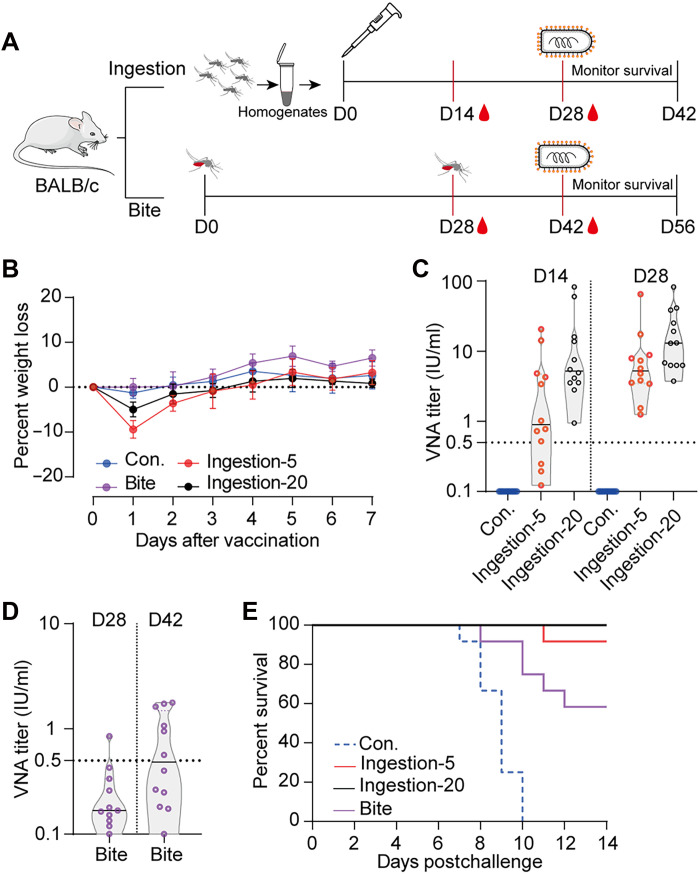
Mosquito-mediated rVSV-RABV vaccination protects mice from RABV challenge. (**A**) Schematic of experimental design of mouse vaccination and challenge studies. Groups of female BALB/c mice (*n* = 12) were vaccinated with rVSV-RABV–carrying mosquitoes either via ingestion (one dose) or bites (two doses). Nonvaccinated mice served as controls (Con.). (**B**) Body weight changes of mice monitored daily for 7 days following the first vaccination. Error bars indicate SD of the mean. (**C**) RABV-neutralizing antibody (VNA) titers in mice vaccinated via ingestion were measured at 14 and 28 days postvaccination in mice orally fed with mosquito homogenates (5 or 20 mosquitoes) using the FAVN test. (**D**) VNA titers in mice vaccinated via bites, i.e., exposed to two mosquito feeding events (30 mosquitoes per exposure) over 4 weeks, at 28 and 42 days post–initial exposure. Above data are representatives of three independent experiments. (**E**) Vaccinated mice were intracerebrally challenged with the RABV CVS-11 strain, and survival was monitored for 14 days.

To enable vaccination in wild environments, we used *Ae. aegypti* as vaccine carriers with two methods of delivery: mosquito ingestion and mosquito bites (Fig. 2A). Mosquitoes were sterilized by 40-Gy x-ray irradiation before viral infection. At 12 days after blood feeding, the viral titer reached 3 × 10^5^ FFU/ml per mosquito. Following immunization by either method, BALB/c mice showed no significant weight loss (Fig. 2B). For the mosquito ingestion method, mice were fed homogenates from either 5 or 20 vaccine-carrying mosquitoes, and virus-neutralizing antibody (VNA) titers were measured using the fluorescent antibody virus neutralization (FAVN) test. By day 14, the VNA geometric mean titers (GMTs) reached 4.6 and 6.8 IU/ml, respectively, both significantly surpassing the protective threshold of 0.5 IU/ml (*34*) against wild-type RABV (Fig. 2C). By day 28, the VNA GMTs further increased to 5.4 IU/ml (range, 3 to 65) and 15 IU/ml (range, 6.3 to 82.6), respectively (Fig. 2C). In the mosquito-bite group, mice were exposed twice to 30 vaccine-carrying mosquitoes. At 28 days after the first exposure, 11 of the 12 mice seroconverted, although their VNA GMTs remained below the threshold. However, after two exposures, VNA GMTs reached 0.5 IU/ml (Fig. 2D).

The mice were subsequently challenged intracerebrally with the lab-adapted RABV strain (CVS-11), and their survival was monitored for 14 days. All control mice succumbed to the virus. By contrast, the groups that ingested 5 and 20 mosquitoes achieved survival rates of 91.6 and 100%, respectively, and the mosquito-bite group achieved a survival rate of 58% (Fig. 2E). These results demonstrate that mosquito-mediated rVSV-RABV vaccination, through either ingestion or bites, elicited robust immune responses in mice and provided effective protection against RABV challenge.

### Mosquito-mediated rVSV-RABV vaccination elicits protective immunity in bats

**Fig. 3.**
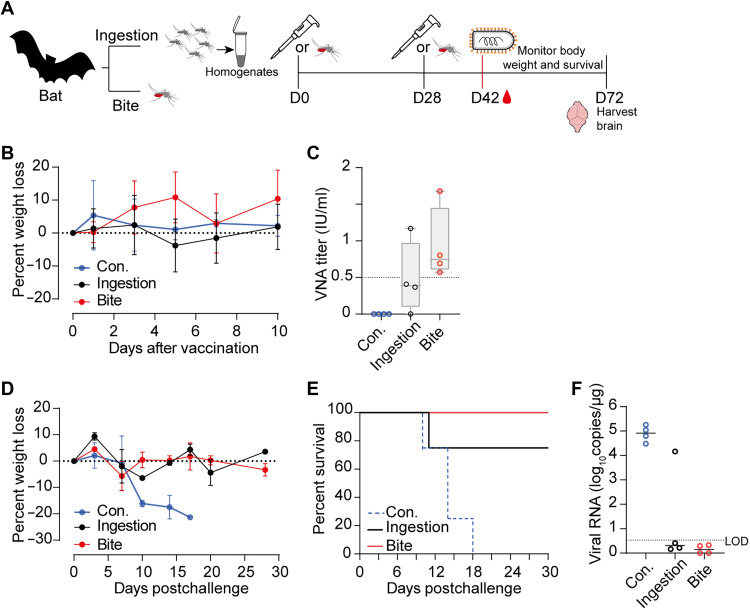
Mosquito-mediated rVSV-RABV vaccination protects bats from RABV challenge. (**A**) Schematic of the experimental design of vaccination and challenge in bats. Groups of bats (*n* = 4) were vaccinated with two doses of rVSV-RABV–carrying mosquitoes, either via ingestion (homogenates from 20 mosquitoes) or bites (30 mosquitoes per dose). Nonvaccinated bats served as controls (Con.). (**B**) Body weight changes of bats monitored for 10 days following the first vaccination. (**C**) RABV-neutralizing antibody (VNA) titers in bats measured at 42 days postvaccination. (**D** to **F**) Bats were intracranially challenged with 1000 MICLD_50_ of the RABV CVS-11 strain at 42 days postvaccination. Body weight changes (D) and survival rates (E) were monitored for 30 days following the challenge. (F) Viral RNA loads in brain tissue quantified by qRT-PCR at 30 days postchallenge. Dotted lines in (F) indicate the detection limit of the assay. Error bars in (B) and (D) indicate SD of the mean.

Given that insectivorous bats are the primary reservoirs of RABV, we further assessed the immunogenicity and protective efficacy of rVSV-RABV in the greater tube-nosed bat (*M. leucogaster*), a natural host of the Irkut virus (*35*). Before immunization, no VNAs were detected in the experimental bats. Two doses of rVSV-RABV were applied with a 28-day interval, via either mosquito ingestion or bites (Fig. 3A). There were no significant changes in body weight following immunization (Fig. 3B). At 14 days after the second exposure, all bats in the mosquito-bite group developed VNA levels exceeding 0.5 IU/ml, whereas only one bat in the ingestion group reached this threshold (Fig. 3C).

Given the lower sensitivity of *M. leucogaster* bats to CVS-11 strain, lethality within 2 weeks was only achieved using a dose of 1000 median lethal dose (LD_50_), measured on mice (MICLD_50_) (fig. S4). Therefore, 6 weeks postvaccination, the bats were intracranially challenged with 1000 MICLD_50_ of RABV. The body weight of bats in both vaccinated groups remained stable following the challenge, whereas the control group experienced 20% weight loss within 18 days (Fig. 3D). The survival rate was 75% in the ingestion group and 100% in the bite group (Fig. 3E). All bats that succumbed to the infection showed clear signs of RABV, with quantitative analysis confirming the presence of the virus in the brain (Fig. 3F), indicating that the cause of death was viral infection.

### Efficacy of oral vaccination against rabies in mice and bats

**Fig. 4.**
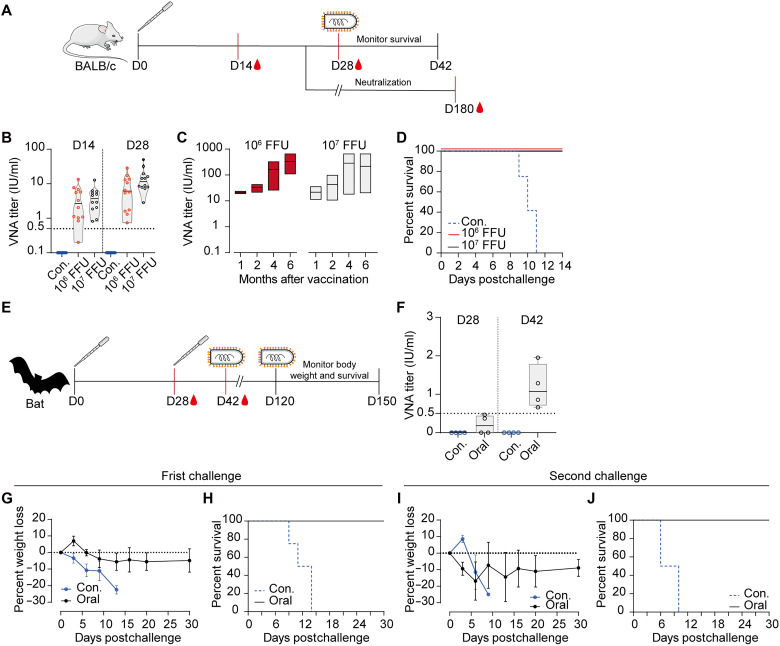
Protective immunity following oral rVSV-RABV vaccination in mice and bats. (**A**) Schematic of experimental design of the vaccination and challenge protocols for mice. (**B** to **D**) Female BALB/c mice (*n* = 12) received a single oral dose of rVSV-RABV (10^6^ or 10^7^ FFU per animal). Mice fed with PBS served as controls (Con.). (B) VNA titers measured at 14 and 28 days postvaccination using the FAVN test. (C) Long-term VNA levels tracked over 6 months. (D) Mice were intracerebrally challenged with 50 LD50 of the RABV CVS-11 strain, and survival rates were recorded for 14 days. Above data are representatives of three independent experiments. (**E**) Schematic of experimental design of the vaccination and challenge protocols for bats. (**F** to **J**) Bats (*n* = 4) were orally vaccinated with two doses of rVSV-RABV (10^7^ FFU per animal). Bats fed with PBS served as controls (Con.). (F) VNA titers in bats measured at 28 and 42 days postvaccination. [(G) to (H)] Bats were intracranially challenged with 1000 MICLD50 of the RABV CVS-11 strain. Body weight (G) and survival rates (H) monitored for 30 days postchallenge. [(I) and (J)] A secondary challenge in bats was conducted 3 months after the primary challenge. (I) Body weight changes following the second challenge. (J) Survival rates recorded for 30 days post–secondary challenge. Error bars in (G) and (I) indicate SD of the mean.

For environments in which mosquito deployment is impractical, we designed an oral vaccination method using saline-based formulations on the basis of the innate mineral-seeking behavior of bats. The efficacy of this approach was first evaluated in mice via oral vaccination with rVSV-RABV at a single dose of 10^6^ or 10^7^ FFU (Fig. 4A). At 14 and 28 days postvaccination, the VNA titers were 2.37 and 3.24 IU/ml, respectively, for the 10^6^ group and 5.31 and 12.16 IU/ml, respectively, for the 10^7^ group (Fig. 4B). VNA levels exceeded 100 IU/ml by 4 months postimmunization, and this level was sustained for up to 6 months (Fig. 4C). To assess the protective efficacy, mice were intracerebrally challenged with 50 LD_50_ of RABV 6 weeks after immunization. All control mice succumbed within 11 days, whereas all vaccinated mice survived for at least 14 days, demonstrating complete protection (Fig. 4D).

For bats, a two-dose regimen of 10^7^ FFU was administered (Fig. 4E). The VNA GMTs remained below 0.5 IU/ml 28 days after the initial immunization; however, it increased to 1.3 IU/ml within 14 days following a booster dose (Fig. 4F). All four vaccinated bats were completely protected against the lethal rabies challenge, whereas the control group succumbed within 15 days (Fig. 4, G and H). Given the likelihood of repeated RABV exposure in wild bat populations, a second challenge was performed 3 months after the first. All vaccinated bats survived for an additional 30 days post–secondary challenge with minimal weight loss (Fig. 4, I and J).

To assess the safety of repeated dosing under potential field application, bats were orally immunized with rVSV-RABV every 2 days for a total of five doses. No significant weight loss was observed, and the VSV RNA was undetectable in most throat swabs, fecal swabs, and all examined organs, indicating a favorable safety profile for repeated administration (fig. S5).

### The efficacy of rVSV-NiV administered via mosquito bite and oral vaccination

NiV is an emerging bat-borne zoonotic virus transmitted by frugivorous bats, and it poses a serious threat to human and domestic animal health. Given that frugivorous bats do not feed on mosquitoes, we proposed a combined vaccine delivery strategy involving mosquito bites and oral administration. Similar to rVSV-RABV, the recombinant virus rVSV-NiV was successfully constructed and rescued, achieving a peak titer of 10^7^ FFU/ml in Vero cells (fig. S6, A and B). The presence of the NiV G protein in the purified virions was confirmed by Western blot (fig. S6C). Additionally, rVSV-NiV infection was detected in both whole mosquitoes and salivary glands (fig. S6D).

**Fig. 5.**
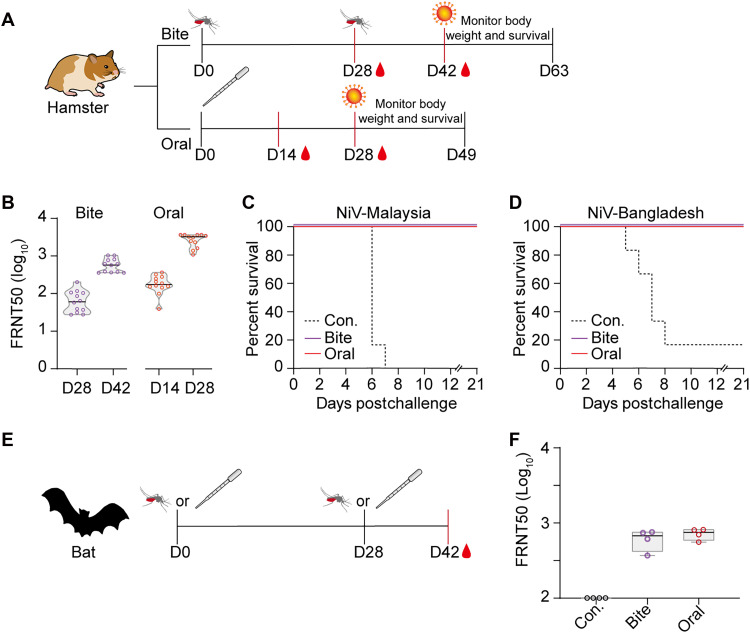
Efficacy of the rVSV-NiV vaccine in hamsters and bats. (**A**) Schematic representation of the experimental design of the vaccination and challenge protocols for golden Syrian hamsters. (**B**) Hamsters (*n* = 12) were exposed twice, at a 4-week interval, to bites from rVSV-NiV–carrying mosquitoes (30 mosquitoes per animal), or were orally given a single dose of rVSV-NiV (10^6^ FFU). Neutralizing antibody (NAb) titers against rVSVΔG-eGFP-NiV F/G were measured at indicated days postvaccination using the focus reduction neutralization test (FRNT). (**C** and **D**) Vaccinated and control hamsters were intraperitoneally challenged with 1000 LD_50_ of NiV (Malaysia or Bangladesh strain). Survival rates were monitored daily for 21 days. Nonvaccinated hamsters served as controls (Con.) (**E**) Schematic representation of the experimental design of the vaccination protocols for bats. (**F**) Bats (*n* = 4) were vaccinated either by exposure to bites from rVSV-NiV–carrying mosquitoes (30 mosquitoes per bat) or by oral administration of rVSV-NiV on two occasions, with an interval of 4 weeks. NAb titers were measured as described in (B). Control bats were nonvaccinated (Con.).

The efficacy of rVSV-NiV was initially evaluated in golden Syrian hamsters, a widely used animal model for NiV studies. After two mosquito bite exposures, hamsters developed high neutralizing antibody (NAb) levels (GMT of 553.4). Similarly, a single oral dose of rVSV-NiV elicited NAb titers of 172 on day 14, which increased to 2406 by day 28 (Fig. 5, A and B). Hamsters were subsequently challenged intraperitoneally with a lethal dose (1000 LD_50_) of either the Malaysia or Bangladesh strain. All vaccinated hamsters survived the challenge and maintained relatively stable body weight postinfection (Fig. 5, C and D, and fig. S7). By contrast, control animals succumbed to NiV infection within 5 to 8 days postchallenge, except for one hamster that survived Bangladesh strain infection.

The efficacy of rVSV-NiV was also evaluated in *M. leucogaster* using the same two vaccination methods (Fig. 5E). At 14 days after the booster dose, GMTs reached 597 in the mosquito-bite group and 711 in the oral vaccination group (Fig. 5F). These results highlight the significant potential of the rVSV-vectored vaccines for frugivorous bat-borne viruses, whether delivered via mosquito bites or oral administration.

### Bat-mosquito interactions in a Guangdong cave

**Fig. 6.**
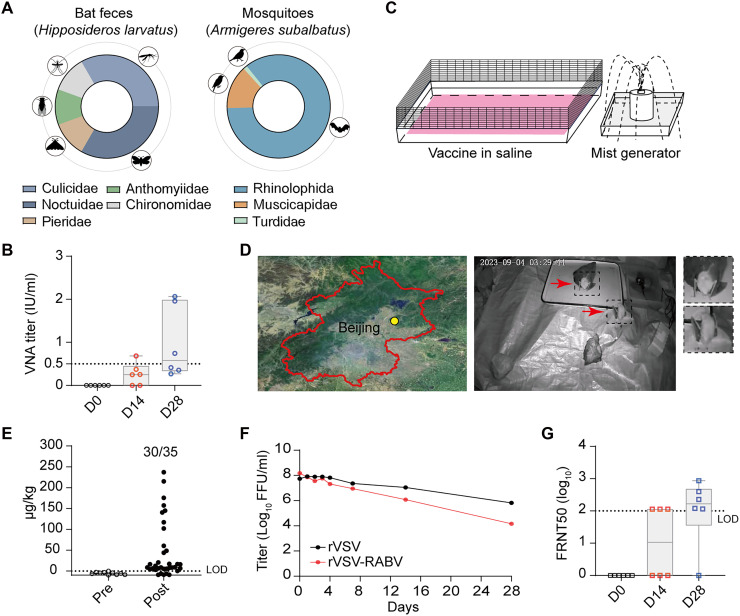
Bat behavior in the field and bat immunization under simulated field conditions. (**A**) Dietary composition of bats (*H. larvatus*) (left) and mosquitoes (*A. subalbatus*) (right) from the same cave in Guangdong province, categorized at the family level using DNA metabarcoding. Data are presented as relative read abundance with different colors representing distinct taxa. (**B**) Six bats were exposed to 300 rVSV-RABV–carrying mosquitoes in a controlled laboratory setting for 48 hours. VNA titers in bats were measured at 0, 14, and 28 days postexposure using the FAVN method. (**C**) Illustration of the saline trap design, comprising a container holding a mixture of vaccine and saline alongside a salt-mist generator. (**D**) The field study site in Beijing, China, is indicated by the yellow circle (left). Surveillance footage shows two wild bats (arrows) drinking from a saline trap placed in the mineral cave (right). (**E**) Detection of tetracycline residues in bat feces by liquid chromatography–tandem mass spectrometry. A saline trap containing tetracycline was deployed in a mineral cave for 1 week. Fresh bat feces were subsequently collected and analyzed for tetracycline residues (Post). Bat feces collected before experiments were set as negative control (Pre). (**F**) rVSV and rVSV-RABV were incubated at 25°C for 28 days, and viral titers were measured by focus-forming assay and expressed as focus-forming units (FFU)/mL. These data are representative of three independent experiments. (**G**) The saline trap containing rVSV-SARS vaccine was placed in a room with six free bats. NAb titers against rVSVΔG-eGFP-SARS were measured at 0, 14, and 28 days postvaccination using the FRNT. Dotted lines in (G) indicate the detection limit of the assay. LOD, limit of detection.

Numerous studies indicate that insectivore bats prey on mosquitoes, while mosquitoes feed on bat blood, supporting the feasibility of mosquito-mediated vaccination (*25*–*28*). Cohabitation of bats and mosquitoes is common in the caves as observed in a cave in Guangdong province (fig. S8). The bat species was identified as *Hipposideros larvatus*, an insectivorous bat species. DNA metabarcoding of their fecal samples revealed a diet consisting of insects from the families Culicidae, Noctuidae, Pieridae, Anthomyiidae, and Chironomidae. Mosquitoes collected from the same cave were classified as *Armigeres subalbatus*, a species widely distributed across China. DNA analysis of blood meals from these mosquitoes identified genetic material from species belonging to the families Rhinolophidae (bats), Muscicapidae, and Turdidae (birds) (Fig. 6A and table S1), confirming that bats are a source of blood meals for these mosquitoes.

### Field simulation of bat immunization strategies

Because of the challenges of conducting experiments in natural environments, we performed field simulation experiments under controlled laboratory conditions. Six insectivorous bats, including three *M. leucogaster* and three *Rhinolophus ferrumequinum*, were confirmed to lack preexisting rabies-neutralizing antibodies before the experiment. The bats were exposed to rVSV-RABV–carrying mosquitoes (~40 mosquitoes/m^3^), allowing for natural biting or ingestion. After 48 hours, ~50% of the mosquitoes were engorged, whereas 15% were unaccounted for, likely due to predation by the bats. VNA levels in both bat species were measured at 14 and 28 days postexposure, reaching 0.36 and 0.69 IU/ml, respectively, with no significant differences between the two species (Fig. 6B).

To enable oral vaccine delivery in wild bat populations, we engineered a saline trap that exploits their innate salt-seeking behavior and acute olfactory capabilities. The trap consisted of a small humidification device generating salt mist to attract bats and a flat-bottom container filled with the vaccine in saline for oral vaccination (Fig. 6C). To evaluate the effectiveness of the trap, we deployed it in a cave with high humidity and added tetracycline (1 mg/liter) to the saline as a biomarker. After 1 week, tetracycline was detected in 85% of fresh bat fecal samples using liquid chromatography–tandem mass spectrometry (Fig. 6, D and E), confirming that the trap successfully attracted wild bats, which ingested the saline. The temperature range of most caves is 15° to 25°C during the active period of bats. The stability of the rVSV vaccine was tested at 25°C and maintained for at least 1 week at 25°C, further demonstrating the feasibility of targeted oral vaccination in wild bats (Fig. 6F).

As bats are also natural hosts of various highly pathogenic coronaviruses, we conducted a controlled laboratory simulation to evaluate the efficacy of a VSV-based SARS-CoV vaccine (rVSV-SARS) in bats (*36*). In an enclosed space (36 m^3^, maintained at 25°C), where six bats (three *M. leucogaster* and three *R. ferrumequinum*) were allowed to fly freely, a saline trap containing rVSV-SARS was placed for 24 hours. Surveillance footage captured multiple drinking events from the trap by both bat species. By day 28, five of the six bats had seroconverted (Fig. 6G).

Our field-simulated experiments demonstrated that robust immunization in wild bats can be achieved through both mosquito-mediated delivery and saline traps, providing a versatile and ecologically integrated strategy for field-based bat vaccination.

## DISCUSSION

Bats occupy a dual position in zoonotic disease ecology: They serve as critical reservoirs for diverse pathogens; yet, they remain vulnerable to population-level threats from some agents (*37*–*39*). Under the “One Health” framework, the development of bat-targeted vaccines is vital for protecting both human health and bat populations. In this study, we leveraged VSV as a vaccine vector and integrated bat and mosquito behaviors to create a vaccine platform suitable for diverse bat species, including insectivorous and frugivorous bats. This innovative approach offers a scalable and efficient solution for immunizing wild bats, addressing critical challenges in disease control and bat conservation.

Mosquitoes are a key food source for insectivorous bats, and they have been identified in the diets of many bat species (*25*, *40*, *41*). Conversely, bat blood has been detected in mosquito blood meals in several studies (*27*, *28*). Our analysis of samples from a cave in Guangdong province further corroborates these interactions, highlighting the potential of mosquitoes as vaccine carriers. The established methodologies for mass-producing and releasing mosquitoes to control arboviruses provide a foundation for deploying mosquito-delivered vaccines to immunize bats on a large scale (*42*, *43*).

As many other wild mammals, bats face dietary sodium limitations and actively seek external mineral sources. We leveraged this behavior to develop an olfactory-driven vaccination system (*29*–*31*), i.e., a saline trap comprising vaccine-laced saline pools and a salt-mist generator. Despite high ambient humidity in roost caves, bats are capable of acute chemosensory detection of sodium gradients, which enables precise localization of saline sources. Field validation demonstrated that this saline trap successfully encouraged drinking behavior in wild bats (Fig. 6, D and E), laying the groundwork for effective oral vaccine delivery.

Wild-type VSV is capable of infecting not only vertebrates but also diverse arthropods. Its natural insect vectors are believed to include mosquitoes, sandflies, blackflies, and midges (*23*). VSV is present in multiple tissues of infected mosquitoes, including salivary glands, enabling bat immunization via mosquito ingestion or bites.

A critical consideration in wildlife vaccination is the trade-off between transmissibility and biosafety. Transmissible vaccines (*44*) offer the potential for high population coverage with minimal input, but they inherently increase evolutionary and ecological risks, including uncontrolled amplification, long-term persistence, and the possibility of reversion to virulence. In contrast, our strategy deliberately prioritizes biosafety through a “limited spread” approach. We used an attenuated rVSV vector (ΔM51) that is incapable of vertical or horizontal transmission within mosquito populations and does not support horizontal shedding in mammals (figs. S2 and S3). Consequently, vaccine exposure remains confined to directly targeted hosts rather than propagating through bat populations.

This containment is further reinforced by multiple biological barriers, including the short life span of laboratory-reared mosquitoes in the wild (~2 weeks) and x-ray sterilization before release. Together, these features prevent sustained circulation of the vaccine in the ecosystem and distinguish this platform from self-disseminating approaches that rely on secondary host-to-host transmission. Despite these advances, translation from laboratory proof of concept to field application requires careful navigation of regulatory and environmental safety considerations.

To address these challenges, we propose a targeted release strategy tailored to specific bat habitats. Based on empirical data indicating that released mosquitoes can disperse up to 300 m horizontally and 100 m vertically (*45*, *46*), deployment grids can be optimized to maximize bat-mosquito encounters while limiting off-site spread. For cave-dwelling insectivorous bats, release cages may be positioned at 100- to 200-m intervals within cave interiors. For tree-roosting frugivorous bats, release stations can be deployed at 50- to 100-m intervals beneath roosting trees, leveraging mosquitoes’ vertical flight capacity.

Additional containment measures can further mitigate regulatory concerns. In semienclosed cave systems, physical barriers such as wind shields installed at cave entrances can restrict mosquito escape while permitting free bat movement. In open environments, particularly near human settlements, spatial repellents containing DEET (N,N-diethyl-meta-toluamide) or pyrethroid-based mosquito coils can be applied around release sites (fig. S9). By integrating biological attenuation with physical and chemical containment, this multilayered framework provides a pragmatic pathway for regulatory evaluation of mosquito-delivered vaccines.

Controlling vaccine dosage in wild bats presents an additional challenge. In our study, administration of five oral doses over 8 days produced no adverse effects in *M. leucogaster* (fig. S5), suggesting that bats can tolerate repeated exposure to rVSV-based vaccines. However, evaluating vaccine effectiveness in natural populations cannot rely on large-scale capture and serological testing of protected species. Accordingly, effectiveness in nature should be assessed using noninvasive, population-level indicators rather than individual immune readouts alone. Our field simulation demonstrated that tetracycline serves as a robust biomarker for estimating vaccine uptake, enabling monitoring through fecal sampling without disturbing bats (Fig. 6E). Beyond exposure assessment, future surveillance should incorporate noninvasive genomic approaches, such as environmental DNA/RNA sampling from roosts to detect reductions in pathogen prevalence over time. This shift toward ecological and population-based metrics aligns more closely with the realities of wildlife vaccination and provides a feasible framework for long-term field evaluation.

The unique characteristics of bat immune systems suggest that existing criteria for protective antibody levels against RABV may not be universally applicable. In our experiments, VNA levels below 0.5 IU/ml conferred protection, aligning with findings from previous studies (*12*, *47*–*49*). Moreover, VSV induces adaptive immune responses beyond NAbs, including T helper cell–mediated immunity. Future evaluations of vaccines should incorporate cellular immune assessments to provide a more comprehensive understanding of protection. While we could not directly assess NiV challenge protection in bats, the NAb levels induced were comparable to protective thresholds observed in hamsters.

High immunogenicity is crucial for wildlife vaccination, as it enables robust protection with minimal doses. Although this study used booster regimens, future efforts will focus on optimizing single-dose formulations, potentially through genetic modifications of mosquitoes to enhance vaccine titers in saliva and improve delivery efficiency.

In conclusion, we developed an integrated strategy using mosquito vectors and saline traps to deliver the rVSV-based vaccines, providing an innovative and scalable approach to immunizing wild bats. Effective vaccination of bats not only mitigates zoonotic spillover risk but also contributes to the conservation of bat biodiversity.

## MATERIALS AND METHODS

### Ethics statement

All animal experiments were conducted in strict accordance with the guidelines outlined in the *Guide for the Care and Use of Laboratory Animals* issued by the Ministry of Science and Technology of the People’s Republic of China. The experimental protocols were reviewed and approved by the Committee on the Ethics of Animal Experiments at the Institute of Zoology, Chinese Academy of Sciences (approval IDs: IOZ-IACUC-2023-077 and IOZ-IACUC-2023-078). All animal studies involving RABV were conducted under ABSL-3 (Animal Biosafety Level 3) conditions. NiV infections were conducted in an ABSL-4 laboratory at Wuhan National Biosafety Laboratory (China Science and Technology Resource: 31120.02.NBL), Chinese Academy of Sciences. Experimental groups consisted of four bats per cohort due to strict ethical quotas for wild-caught protected species (*M. leucogaster* and *R. ferrumequinum*). Field sampling prioritized minimal ecological impact, aligning with IUCN (International Union for Conservation of Nature) guidelines for bat conservation.

### Cells and antibodies

Vero (African green monkey kidney) cells and human embryonic kidney (HEK) 293T cells were obtained from the American Type Culture Collection and maintained in Dulbecco’s modified Eagle’s medium (DMEM) supplemented with 8% fetal bovine serum (FBS), 1% l-glutamine, and 1% penicillin-streptomycin. All cells were maintained at 37°C in a 5% CO_2_ incubator. Rabbit polyclonal anti–NiV G antibody (AtaGenix Laboratories, Wuhan, China) and rabbit polyclonal anti-RABV glycoprotein antibody (Thermo Fisher Scientific, Waltham, MA, USA) were used.

### Construction, rescue, and characterization of recombinant VSV viruses

The plasmid encoding VSV (pVSV) and pVSVΔG–enhanced green fluorescent protein (eGFP) vectors were designed, synthesized, and constructed as described previously (*50*). The coding sequences of the glycoproteins from the RABV PM1503 strain (GenBank, DQ099525.1) and NiV Malaysia strain (GenBank, AF212302.2) were synthesized and inserted between the Mlu I and Not I restriction sites of the pVSV vector plasmid, generating pVSV-RABV and pVSV-NiV plasmids, each retaining the native VSV G gene. For the construction of plasmids lacking the VSV G gene, the coding sequences of the NiV F and G genes were inserted between the Mlu I and Not I sites of the pVSVΔG-eGFP plasmid, resulting in pVSVΔG-eGFP-NiV F/G plasmids, which do not contain the native VSV G gene.

Recombinant VSVs were generated using a reverse genetics approach. HEK293T cells were transfected with the full-length VSV plasmids described above, along with supporting plasmids encoding T7 polymerase and the VSV N, P, M, G, and L proteins, using the calcium phosphate method (*51*). Recombinant viruses were harvested upon the appearance of obvious cytopathological effects and further amplified in Vero cells. Purified virions, obtained via ultracentrifugation, were characterized by Western blot analysis using antibodies specific to the glycoproteins of RABV or NiV.

### Growth kinetics of rVSVs

Vero cells (2 × 10^6^) seeded in T75 flasks were infected with rVSVs at a multiplicity of infection of 0.01. After 3 hours, the inoculum was removed, and the cells were replenished with DMEM supplemented with 2% FBS. Cultures were incubated at 28°C, and aliquots for viral titration were collected every 12 hours. Viral titers were determined using a focus-forming assay.

### Focus-forming assay

Vero cells were seeded in 96-well plates at 10,000 cells per well 24 hours before the experiment. Virus supernatants were serially diluted 10-fold in DMEM with 2% FBS and incubated with cells for 3 hours (100 μl per well). Then, the media with viruses were replaced with fresh DMEM with 2% FBS, 1% penicillin/streptomycin, and 20 mM NH_4_Cl. After incubation at 28°C for 24 hours, the cells were fixed, permeabilized, and blocked. Virus foci were stained with anti-RABVG or anti–NiV G antibody followed by Alexa Fluor 488 goat anti-rabbit secondary antibody (catalog no. A11008, Thermo Fisher Scientific, Waltham, MA, USA) and counted under a fluorescence microscope.

### Mosquito rearing and infection

Lab-adapted *Ae. aegypti* mosquitoes (UGAL/Rockefeller strain) were maintained at 28°C and 80% relative humidity. Larvae were fed a diet of mouse food, albumin, and yeast extract, whereas adults were provided with a solution of water and 10% (w/v) sucrose.

Female mosquitoes aged 6 to 7 days were starved for 20 hours before viral infection. A mixture of virus (400 μl) and mouse blood (400 μl) was preheated at 37°C for 30 min and offered to mosquitoes using a membrane feeder maintained at 37°C. After 30 min, engorged mosquitoes were selected and incubated at 28°C for further experiments.

### Mosquito irradiation

Female mosquitoes aged 3 days were irradiated with 40 Gy using the RS 2000 Series Biological Irradiator (Rad Source Technologies Inc., Buford, Georgia, USA). Survival rates were assessed 3 days postirradiation, and surviving mosquitoes were blood fed with rVSVs. Fully engorged mosquitoes were counted to calculate the engorgement rate. After oviposition, the egg-laying and egg-hatching rates were recorded. Viral titers were quantified in individual whole mosquitoes and in the salivary glands pooled from five mosquitoes.

### Venereal and transovarial transmission

Female mosquitoes aged 3 days were infected with 100 FFU of rVSV through intrathoracic microinjection and subsequently mated with uninfected male mosquitoes. Viral RNA in both the female and male mosquitoes was assessed by quantitative reverse transcription polymerase chain reaction (qRT-PCR) at 10 days postinfection.

In a separate experiment, female mosquitoes aged 3 days were infected with 100 FFU of rVSV via intrathoracic microinjection and mated with uninfected males. After 7 days, the females were blood fed using a membrane feeder, and fully engorged females (F0) were selected. Eggs laid by F0 females were collected, and the offspring (F1) were reared to adulthood. Viral RNA in F0 and F1 mosquitoes was quantified by real-time PCR.

### Bat capture, husbandry, and biosafety

Bats (*M. leucogaster* and *R. ferrumequinum*) used in this study were wild caught from natural cave roosts in the suburban areas of Beijing, China, under permissions granted by the relevant local authorities. After capture, bats were transported to the laboratory facility and quarantined for at least 2 weeks for acclimatization and biosafety observation before experimental procedures. Baseline serum samples and swabs were collected during quarantine to confirm the naïve status of all experimental bats with respect to RABV, NiV, and other relevant zoonotic pathogens. Following quarantine, bats were randomly assigned to experimental groups of four to six animals.

During the study period, bats were housed in mesh cages under controlled environmental conditions, with ambient temperatures maintained at 25° to 30°C and relative humidity above 40%. Animals were fed ad libitum with yellow mealworms (*Tenebrio molitor*) supplemented with vitamins, and fresh water was provided continuously. All bats were monitored daily for general health status and behavior.

To ensure biosafety and biosecurity, bats were clinically examined before inclusion in experimental procedures, and individuals showing signs of illness or abnormal behavior were excluded. Blood samples for serological assays were collected from the saphenous vein of the uropatagium. All experimental manipulations involving live bats were conducted by trained personnel using appropriate personal protective equipment and in compliance with institutional biosafety regulations.

All animal procedures were approved by the Institutional Animal Care and Use Committee and were performed in accordance with relevant guidelines and regulations. Details of specific immunization, challenge experiments, and field simulation studies are described in the corresponding sections below.

### Animal immunization

Female BALB/c mice and golden Syrian hamsters (*Mesocricetus auratus*) (6 to 8 weeks old) were purchased from Beijing Vital River Laboratory Animal Technology (licensed by Charles River Laboratories). Female mosquitoes were irradiated with x-rays at a dose of 40 Gy and subsequently infected with rVSVs through a blood meal. Before immunization, the viral load in the whole body and salivary glands of the mosquitoes was quantified using focus-forming assay at 12 days postinfection. For immunization via oral ingestion, vaccine-carrying mosquitoes (*n* = 5 or 20) were homogenized in 100 μl of PBS at 60 Hz for 90 s using a Tissuelyser (Shanghai Jingxin Industrial Development Co. Ltd., Shanghai, China). The resulting homogenate was administered to the animals via a pipette. For immunization via mosquito bites, the animals were exposed to the vaccine-carrying mosquitoes for 30 min. Mice (*n* = 12) were immunized once, whereas hamsters (*n* = 12) and bats (*n* = 4) received two immunizations with a 4-week interval. Body weight was monitored daily for 7 days following the first vaccination. Serum samples were collected to measure NAb titers against RABV or NiV.

### RABV challenge

BALB/c mice were challenged intracerebrally with 50 LD_50_ RABV (CVS-11 strain) 28 days after the single-dose vaccination or 14 days after the second dose in the two-dose immunization group. Survival was monitored daily for 14 days, after which the mice were euthanized.

To determine the lethal dose of the CVS-11 strain for *M. leucogaster* bats, three groups of bats (*n* = 4) were inoculated intracranially with varying doses of RABV (100, 1000, and 10,000 MICLD_50_). On the basis of the results, a CVS-11 strain dose of 1000 MICLD_50_ was selected, and bats were challenged 14 days after the second vaccination dose. The animals were observed for 30 days for signs of rabies and changes in body weight. At the end of the observation period, bats were euthanized under anesthesia, and brain tissues were collected for RABV RNA detection.

### NiV challenge

Hamsters were challenged with 1000 LD_50_ NiV (Malaysia or Bangladesh) via the intraperitoneal route 14 days after the final vaccination. Weight changes and mortality were monitored for 14 and 21 days postchallenge, after which the hamsters were euthanized.

### VSV infection in pigs

Bama miniature pigs (4 weeks old) were obtained from the Yunnan Animal Science and Veterinary Institute (Yunnan, China). Six pigs received 10^8^ FFU rVSV, whereas three control pigs received DMEM. Both groups were inoculated via intradermal injection at the apex of the snout. Clinical examinations and body weight monitoring were conducted regularly. Swab samples (nasal, throat, and rectal) and blood samples were collected for virological analysis using qRT-PCR. NAb titers against VSV were measured at 14 days postinfection. At 3 days postinfection, three pigs from the rVSV group were euthanized. Tissue samples from the tongue, lip, nose, trachea, brain, heart, liver, spleen, lung, and kidney were collected for virological analysis.

### Neutralization assay

The RABV neutralization assay was conducted in a certified BSL-2 laboratory. Sera were analyzed for VNAs using the FAVN assay, with RABV (CVS-11 strain) as the test virus and BHK-21 cells as the host cells (*52*). The calibrated World Health Organization international standard immunoglobulin (second human rabies immunoglobulin preparation, National Institute for Standards and Control, Potters Bar, Hertfordshire, UK) adjusted to 0.5 IU and naïve serum were included as positive and negative controls, respectively. VNA titers were determined using the Spearman-Kärber method.

For the focus reduction neutralization test (FRNT), 100 to 1000 FFU of rVSV-eGFP, rVSVΔG-eGFP-NiV F/G, or rVSVΔG-eGFP-SARS were incubated with three-fold serial dilutions of heat-inactivated sera at room temperature for 30 min. The virus-serum mixtures were then added to cells seeded in 96-well plates. After 3 hours, the culture medium was removed, and fresh DMEM supplemented with 2% FBS and 20 mM NH_4_Cl was added. Green fluorescent protein–positive cells were counted 20 hours postinfection using the Opera Phenix High-Content Screening System (PerkinElmer, Waltham, MA, USA). Neutralization titers were calculated as the dilution resulting in 50% inhibition of virus infection (FRNT_50_), determined using the Reed-Muench method.

### RNA extraction and qRT-PCR

Total RNA was extracted from homogenized animal tissues and pooled mosquito body parts, using TRIzol Reagent (catalog no. RE703, Thermo Fisher Scientific, MA, USA) following the manufacturer’s instructions. For blood samples and viral culture supernatants, RNA extraction was performed using the TIANamp Virus RNA Kit (catalog no. DP315-R, TIANGEN Biotech Co. Ltd., China). Reactions were performed using the One Step TB Green PrimeScript RT-PCR Kit (catalog no. RR066A, TaKaRa Bio, Shiga, Japan) and Applied Biosystems QuantStudio Real-Time PCR System (Thermo Fisher Scientific, MA, USA). Each sample was tested in triplicate to ensure reliability. The primers used for qRT-PCR were as follows: 5′-CGGAGGATTGACGACTAATGC-3′ and 5′-ACCATCCGAGCCATTCGA-3′ for rVSV and 5′-CGCAAGACAATGTCATATTC-3′ and 5′-AAGAGACATGTCAGACCATA-3′ for RABV. Real-time PCR data were collected and analyzed using QuantStudio 12K Flex software (version 1.4, Applied Biosystems, USA).

### Determination of tetracycline

Fresh bat fecal samples were collected 1 week after deploying saline traps containing tetracycline (1 mg/liter) in field caves. Tetracycline residues in guano were analyzed through extraction using McIlvaine-Na_2_EDTA buffer, purification with a hydrophilic-lipophilic balanced solid-phase extraction column, and quantification by liquid chromatography–tandem mass spectrometry.

### DNA metabarcoding–based diet analysis

Fecal samples from bats were collected in bat cave located in the Guangdong Province, and mosquito samples were obtained from the cave in Guangdong. DNA was extracted using a commercial DNA extraction kit (catalog no. D0063, Beyotime Biotech Inc., Shanghai, China). PCR amplification was performed with PrimeSTAR HS DNA Polymerase with GC Buffer (catalog no. R044Q, TaKaRa Bio, Shiga, Japan). The following primers were used: 5′-CCIGAYATRGCITTYCCICG-3′ and 5′-TANACYTCNGGRTGNCCRAARAAYCA-3′ for the cytochrome c oxidase subunit *I* (COI) barcode region and 5′-TAGAACAGGCTCCTCTAG-3′ and 5′-TTAGATACCCCACTATGC-3′ for 12*S* ribosomal DNA . Dual-index barcodes were added to all primers. The purified amplicons were pooled and subjected to paired-end sequencing (PE150 or PE250) on an Illumina platform (Novogene Bioinformatics Technology Co. Ltd., Beijing, China).

### Field experiment

For mosquito-mediated vaccination, six bats were allowed to fly freely within an enclosed space (2.2 m by 2 m by 1.7 m). A total of 300 mosquitoes carrying the rVSV-RABV vaccine were released and recaptured 48 hours later. Blood samples were collected on days 14 and 28 postvaccination to evaluate VNA titers.

For oral liquid vaccination, another group of six bats was housed in a separate enclosure (5 m by 3.2 m by 2.3 m), in which a saline trap containing the rVSV-SARS vaccine was placed for 24 hours. Blood samples were collected on days 14 and 28 postvaccination to assess NAb titers against rVSVΔG-eGFP-SARS.

### Determination effects of mosquito repellent and electrothermal mosquito coil

One hundred 3-day-old lab-adapted *Ae. aegypti* mosquitoes were housed in cages size measuring 40 cm by 30 cm by 30 cm. Four testers participated, with a 50 mm by 50 mm area of skin on each hand exposed. One hand was evenly coated with repellent containing 15% DEET at a concentration of 1.5 μl/cm^2^, while the remaining part of the hand was securely covered, and the other hand served as an untreated control. After 1 hour, the treated and control hands were alternately inserted into a mosquito cage with adequately aggressive mosquitoes for 2 min. Mosquitoes landing and attempting to blood-feed were observed. The effective protection time of DEET was determined through hourly tests conducted for up to 5 hours.

One hundred *Ae. aegypti* mosquitoes were released into a confined chamber (3 m by 2.5 m by 3 m). Once the mosquitoes resumed typical activity, electrothermal mosquito coils containing 1.2% meperfluthrin were placed at the center of the chamber and activated. After 1 hour, the deceased mosquitoes were collected using mosquito cages, and the knockdown rate was calculated to evaluate the effectiveness of the coils.
