# Supplementary material for: Ecological vaccination: A strategy to prevent zoonotic spillover from bats
Source: Sci Adv. 2026 Mar 11;12(11):eaec0269. doi: 10.1126/sciadv.aec0269 (PMC12978229; doi:10.1126/sciadv.aec0269)
Supplement: Supplementary file 1 — Figs. S1 to S9 Table S1 [file sciadv.aec0269_sm.pdf]

Science Advances
AAAS

Supplementary Materials for

Ecological vaccination: A strategy to prevent zoonotic spillover from bats

Hongyue Li *et al.*

Corresponding author: Chao Shan, shanchao@wh.iov.cn; Aihua Zheng, zhengaihua@ioz.ac.cn

Sci. Adv. **12**, eaec0269 (2026)
DOI: 10.1126/sciadv.aec0269

This PDF file includes:

Figs. S1 to S9
Table S1

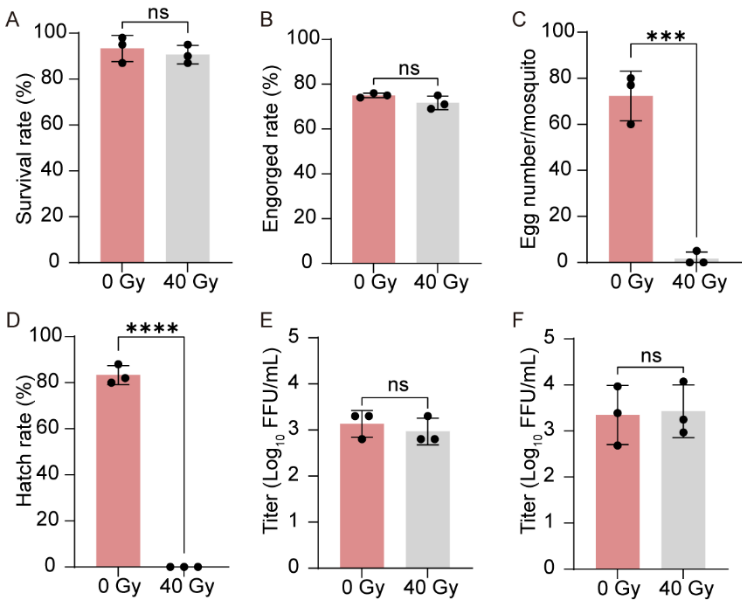

Fig. S1 Effects of X-ray irradiation on *Ae. aegypti* infected with rVSV

Female *Ae. aegypti* mosquitoes were blood-fed with rVSV at 3–4 days post-X-ray irradiation. The following parameters were evaluated and compared between irradiated and non-irradiated mosquitoes: survival rate (**A**), engorgement rate (**B**), number of eggs laid per mosquito (**C**), hatching rate of eggs (**D**), viral titer in whole mosquitoes (**E**), and viral titer in pooled saliva glands from five mosquitoes (**F**). Error bars indicate standard deviation of the mean. *P*-values were determined using the two-sided unpaired *t*-test. ns indicates nonsignificance, ***$P < 0.001$, ****$P < 0.0001$. Above data are representatives of three independent experiments.

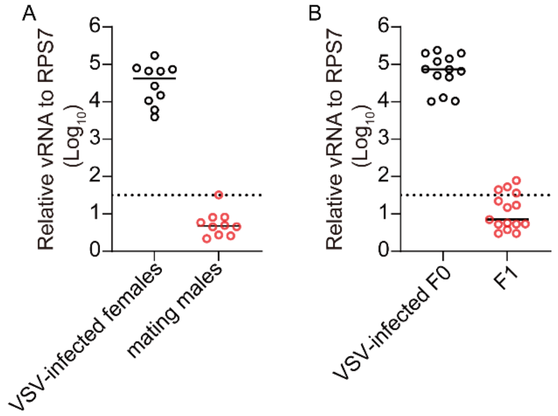

Fig. S2 Venereal and transovarial transmission potential of rVSV in *Ae. aegypti*

(**A**) Relative viral RNA levels in infected female *Ae. aegypti* mosquitoes and mating males after copulation (n = 10). (**B**) Relative viral RNA levels in infected female mosquitoes (F0) and their offspring (F1). The dotted line indicates the detection limit. Above data are representatives of three independent experiments.

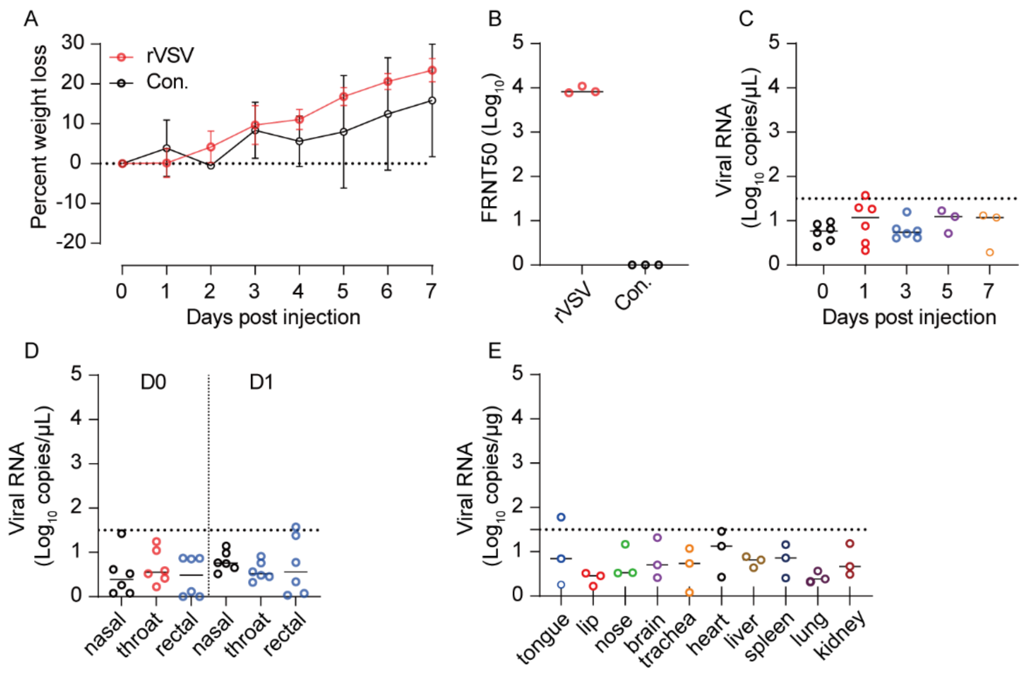

Fig. S3 Safety evaluation of rVSV in Bama miniature pigs

Bama miniature pigs were inoculated with rVSV or DMEM (Con.) via intradermal injection at the apex of the snout. (**A**) Body weight changes in pigs inoculated with 10^8 FFU of rVSV (*n* = 6) compared to mock-inoculated pigs (*n* = 3). Error bars indicate standard deviation of the mean. (**B**) $FRNT_{50}$ values of pig serum at 14 days post-inoculation against rVSV-eGFP. (**C**) Viral RNA levels in blood at indicated time points post-inoculation, as determined by qRT-PCR. (**D**) Viral RNA levels in swab samples (nasal, throat, and rectal) at days 0 (D0) and 1 (D1) post-inoculation. (**E**) Viral RNA levels in various tissues from three pigs euthanized on day 3 post-inoculation. Error bars indicate standard deviation of the mean. The dotted line indicates the detection limit (**C, D, E**).

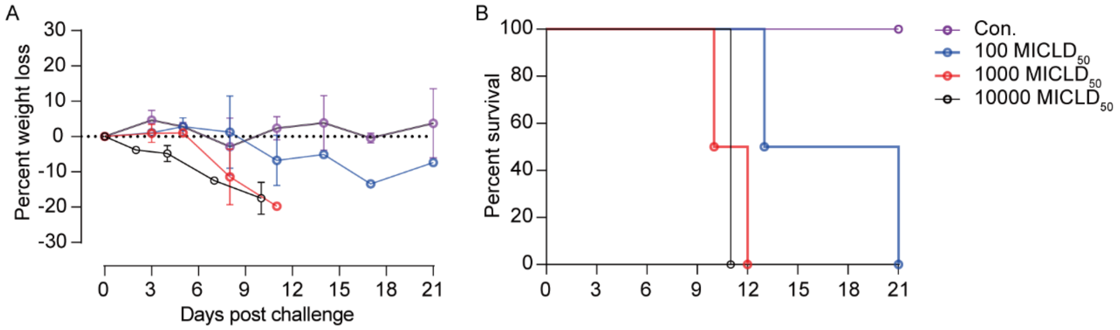

Fig. S4 Susceptibility of *Murina leucogaster* bats to the RABV CVS-11 strain

Groups of *Murina leucogaster* bats (n = 4) were intracranially challenged with 100–10,000 MICLD$_{50}$ of the RABV CVS-11 strain. (**A**) Body weight changes and (**B**) mortality rates were monitored for up to 21 days post-challenge. The control group received PBS (Con.). Error bars indicate standard deviation of the mean.

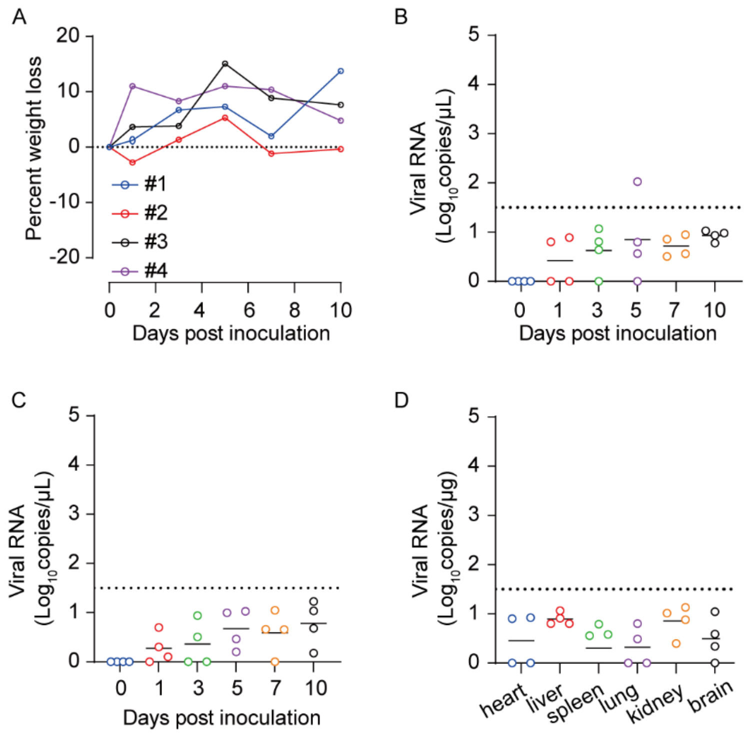

Fig. S5 Safety profile of rVSV-RABV in bats

Four bats were orally administered rVSV-RABV (10^7 FFU/animal) on days 0, 2, 4, 6, and 8. (**A**) Body weight was recorded for 10 days. (**B-C**) Viral RNA levels in throat swabs (**B**) and fecal swabs (**C**) were quantified using qRT-PCR at various time points. (**D**) At 10 days post-inoculation, bats were euthanized, and tissue samples were collected for viral RNA analysis using qRT-PCR. The dotted line (**B, C**) indicates the detection limit.

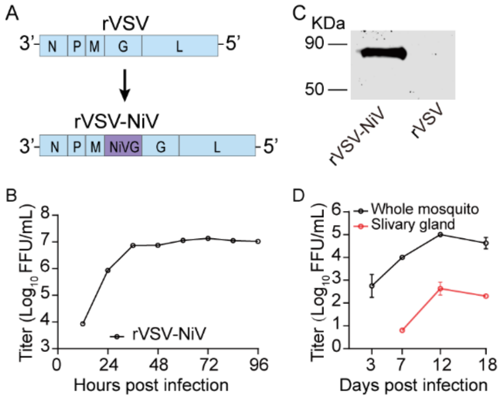

Fig. S6 Characterization of rVSV-NiV and its propagation in *Aedes aegypti*

(**A**) Schematic diagram of rVSV-NiV genomic organization. The open reading frame of the NiV *G* gene was inserted between the VSV matrix (*M*) and glycoprotein (*G*) genes. (**B**) Growth kinetics of rVSV-NiV in Vero cells. (**C**) Western blot analysis of rVSV-NiV and rVSV purified from supernatants, probed with anti-NiV G protein antibodies. (**D**) *Ae. aegypti* were blood-fed with rVSV-NiV. Viral titers in whole mosquitoes or salivary glands were determined by a focus-forming assay. Error bars indicate standard deviation of the mean. Above data are representatives of three independent experiments.

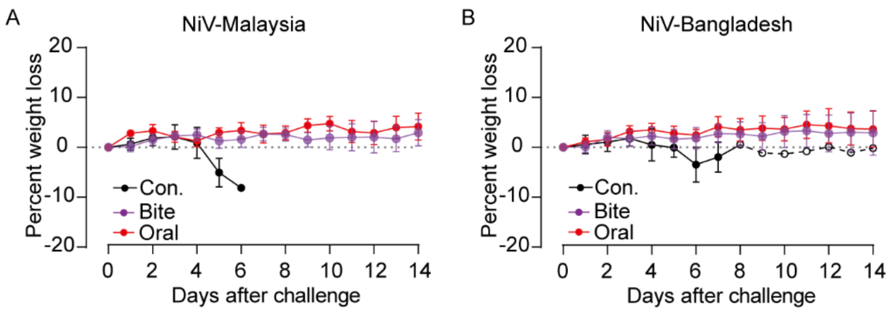

Fig. S7 Weight changes in hamsters post-NiV challenge

Hamsters (n = 12) vaccinated via mosquito bites or oral inoculation were intraperitoneally challenged with 1000 LD_{50} of Nipah virus (Malaysia or Bangladesh strain). Body weight was monitored daily for 14 days post-challenge. Non-vaccinated hamsters served as controls (Con.). Error bars represent the standard deviation of the mean.

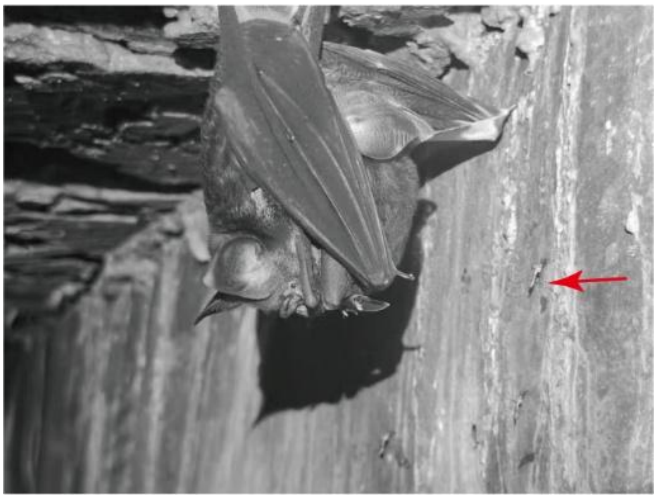

Fig. S8 Photo of *Hipposideros larvatus* bats and *Armigeres subalbatus* mosquitoes (arrow) in a cave in Guangdong province

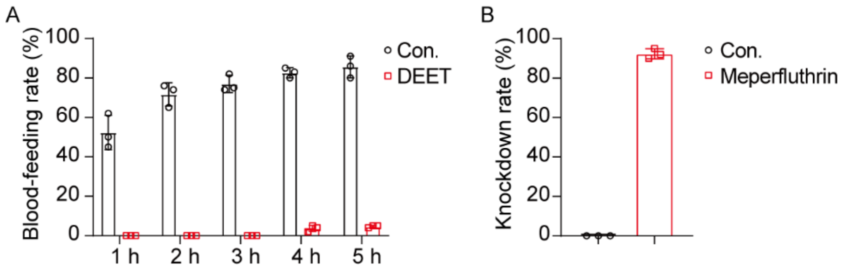

Fig. S9 Efficacy of DEET-based repellents and electrothermal mosquito coils

(**A**) Blood-feeding rates of *Ae. aegypti* were assessed by inserting either a hand coated with a 15% DEET-based repellent or an untreated hand (Con.) into a mosquito cage containing 100 aggressive mosquitoes for two minutes. (**B**) *Ae. aegypti* were released into a sealed chamber containing an activated electrothermal mosquito coil with 1.2% meperfluthrin. The knockdown rate of mosquitoes was recorded 1 h after exposure. The control group (Con.) was left untreated. Above data are representatives of three independent experiments.

Table S1 Diet analysis of bats (*Hipposideros larvatus*) and mosquitoes (*Armigeres subalbatus*) in Guangdong province.

Samples	Species	Family
Bat feces	*Armigeres subalbatus*	*Culicidae*
	Anopheles sinensis	*Culicidae*
	Macropelopia grandivolsella	*Chironomidae*
	Hydrillodes lentalis	*Noctuidae*
	Spodoptera mauritia	*Noctuidae*
	Pieris rapae	*Pieridae*
	Anthomyiidae	*Anthomyiidae*
Mosquitoes	*Hipposideros larvatus*	*Rhinolophida*
	Turdus merula	*Turdidae*
	Muscicapa dauurica	*Muscicapidae*
